# Fine mapping and marker development for the wheat leaf rust resistance gene *Lr32*

**DOI:** 10.1093/g3journal/jkac274

**Published:** 2022-10-18

**Authors:** Jyoti Saini Sharma, Curt A McCartney, Brent D McCallum, Colin W Hiebert

**Affiliations:** Agriculture and Agri-Food Canada, Morden Research and Development Centre, Morden, MB R6M 1Y5, Canada; Department of Plant Sciences, University of Manitoba, Winnipeg, MB R3T 2N2, Canada; Agriculture and Agri-Food Canada, Morden Research and Development Centre, Morden, MB R6M 1Y5, Canada; Agriculture and Agri-Food Canada, Morden Research and Development Centre, Morden, MB R6M 1Y5, Canada

**Keywords:** *Lr32*, leaf rust, wheat, Plant Genetics and Genomics

## Abstract

Wheat leaf rust is caused by the fungal pathogen *Puccinia triticina* and is one of the wheat diseases of concern globally. Among the known leaf rust resistance genes (Lr) genes, *Lr32* is a broadly effective gene derived from the diploid species *Aegilops tauschii* coss. accession RL5497-1 and has been genetically mapped to chromosome arm 3DS. However, *Lr32* resistance has not been utilized in current cultivars in part due to the lack of modern, predictive DNA markers. The goals of this study were to fine map the *Lr32* region and develop SNP-based kompetitive allele-specific polymerase chain reaction markers. The genomic analysis was conducted by using doubled haploid and F_2_-derived mapping populations. For marker development, a 90K wheat chip array, 35K and 820K Axiom R SNPs, *A. tauschii* pseudomolecules v4.0 and International Wheat Genome Sequencing Consortium ReqSeq v2.1 reference genomes were used. Total 28 kompetitive allele-specific polymerase chain reaction and 2 simple sequence repeat markers were developed. The *Lr32* region was fine mapped between kompetitive allele-specific polymerase chain reaction markers *Kwh142* and *Kwh355* that flanked 34–35 Mb of the diploid and hexaploid reference genomes. Leaf rust resistance mapped as a Mendelian trait that cosegregated with 20 markers, recombination restriction limited the further resolution of the *Lr32* region. A total of 10–11 candidate genes associated with disease resistance were identified between the flanking regions on both reference genomes, with the majority belonging to the nucleotide-binding domain and leucine-rich repeat gene family. The validation analysis selected 2 kompetitive allele-specific polymerase chain reaction markers, *Kwh147* and *Kwh722*, for marker-assisted selection. The presence of *Lr32* along with other Lr genes such as *Lr67* and *Lr34* would increase the resistance in future wheat breeding lines and have a high impact on controlling wheat leaf rust.

## Introduction

Leaf rust is the most common and widely occurring wheat rust disease (*Triticum aestivum* L. 2*n* = 6*x* = 42, AABBDD) and is caused by a biotrophic fungal pathogen *Puccinia triticina* Eriks (*Pt*) (Bolton et al. 2008). There are presently 79 leaf rust resistance (Lr) genes that have been identified (McIntosh *et al.* 2019). Among 3 wheat genomes, the D genome progenitor *Aegilops tauschii* coss. (2*n* = 2*x* = 14, DD genome) is known to be a great resource of rust resistance genes compared with other 2 progenitors *T. urartu* Tumanian ex Gandylian (2*n* = 2*x* = 14, AA) and a close relative of *Aegilops speltoides* ssp*. lingustica* Tausch (2*n* = 2*x* = 14, SS) (see Faris 2014 for review). To date, 5 Lr genes have been transferred to bread wheat from the *A*. *tauschii*: *Lr21*/*Lr40* (1DS), *Lr22a* (2DS), *Lr32* (3DS), *Lr39*/*Lr41* (2DS), and *Lr42* (1DS) (Dyck and Kerber 1970; Rowland and Kerber 1974; Kerber 1987; Cox *et al.* 1994).

Plant disease resistance genes have been classified into race-specific and nonrace-specific groups. The race-specific resistance follows the gene-for-gene model described by Flor (1955) that states that for each resistance (R) gene in the plant there will be a corresponding avirulence (AVR) gene in the pathogen. Some of the nonrace-specific resistance type genes are effective against many pathogens and were reported to be associated with a variety of genes encoding receptor kinases, transcription factors, membrane proteins, Kinase-START, and ATP-binding cassette (ABC) transporters (Singh *et al.* 2015). Many of the race-specific R genes in wheat were associated with the nucleotide-binding domain and leucine-rich repeat (NLR) gene family. To date, only 7 Lr genes have been cloned *Lr1* (Cloutier *et al.* 2007), *Lr10* (Feuillet *et al.* 2003), *Lr21* (Huang *et al.* 2003), *Lr22a* (Thind *et al.* 2017), *Lr34*/*Yr18*/*Sr57*/*Pm38* (Krattinger *et al.* 2009), *Lr67*/*Yr46*/*Sr55*/*Pm46* (Moore *et al.* 2015), and *Lr14a* (Kolodziej *et al.* 2021). Out of which, race-specific leaf rust genes *Lr1*, *Lr10*, and *Lr21* belong to the NLR gene family. *Lr14a* was sequenced and was found to belong to ankyrin (ANK) transmembrane-like gene family, which is a first in wheat (Kolodziej *et al.* 2021). Genes conditioning race-specific resistance tend to lose their effectiveness as the pathogen population evolves virulence. For example, the *P. triticina* population in Canada has evolved virulence to *Lr1*, *Lr2a*, *Lr10*, *Lr12*, *Lr13*, *Lr14a*, and *Lr21* which were all used in Canadian wheat cultivars to control leaf rust (McCallum *et al.* 2016). In contrast durable, race nonspecific, slow-rusting leaf rust resistance genes *Lr34*, *Lr46*, and *Lr67* have proved to be long-lasting and are able to confer resistance against multiple pathogens and races (Singh *et al.* 1998; Krattinger *et al.* 2009; Moore *et al.* 2015; McCallum *et al.* 2016). High levels of durable leaf rust resistance have been achieved by combining race-specific and race-nonspecific resistance genes in the same cultivar, such as the Canadian cultivar Carberry with *Lr2a*, *Lr16*, *Lr23*, *Lr34*, and *Lr46* (Bokore *et al.* 2022). Consequently, new gene combinations need to be introduced in the wheat cultivars to achieve the durable resistance and decrease the selection pressure on *Pt* populations for virulence against R genes. For combining multiple R genes, the availability of gene-based and functional molecular makers is ideal. Within the last few years, high-throughput genetic analysis has become more advanced with the development of single nucleotide polymorphism (SNP) chips (Wang *et al.* 2014; Allen *et al.* 2017) and the availability of reference genome resources (Luo *et al.* 2017; Maccaferri *et al.* 2019; Zhu *et al.* 2019) which has led to better markers for a higher number of genes and resources needed for gene cloning experiments.

Among the broadly effective leaf rust resistance genes (*Lr21*, *Lr22a*, *Lr32*, *Lr52*, and *Lr60*) identified at the Morden Research and Development Centre, AAFC, Morden (and the former Cereal Research Centre, Winnipeg): *Lr32*, *Lr52*, and *Lr60* have not been deployed in Canadian commercial cultivars (Thomas *et al.* 2010) and *Lr22a* has been deployed in 3 cultivars (AC Minto, 5500HR, and 5600HR) that occupied minimal acreage (Hiebert *et al.* 2007). The seedling leaf rust resistance gene *Lr32* was discovered in *A. tauschii* coss. (accession number RL5497-1) and was mapped using simple sequence repeat (SSR) markers on chromosome arm 3DS. It was physically located in deletion bin 6 (3DS6–0.55–1.00) which spans ∼75 cM of genetic distance (Kerber 1987, 1988; Sourdille *et al.* 2004; Thomas *et al.* 2010). The SSR map was not dense, and while some SSRs were very closely linked to *Lr32*, markers that reliably flanked the gene had large genetic distances to *Lr32*. Hence, the objectives of this study were: (1) to develop the new kompetitive allele-specific polymerase chain reaction (PCR) (KASP) markers for *Lr32* suitable for marker-assisted selection (MAS) and (2) fine map the *Lr32* region.

## Materials and methods

### Plant material

Wheat line BW196 (=Katepwa*6//RL5713/2*MarquisK) was found to be heterogeneous for *Lr32* based on phenotype and molecular marker data (Thomas *et al.* 2010). Therefore, for the development of mapping populations, a single resistant reselection (BW196R) was used as a resistance parent as explained in Thomas *et al.* (2010). A double haploid (DH) population (*n* = 244), an initial F_2_ (*n* = 196) population, and an expanded large F_2_ (*n* ≈ 2,000) population were developed from a cross between the susceptible parent Thatcher and the resistant line BW196R. The initial, smaller F_2_ population and the expanded F_2_ population were generated from the same cross, but there was no overlap in individuals between the 2 populations. The F_3_ families were developed from the large F_2_ population lines based on markers flanking the *Lr32* region (recombinants were selected, detailed description in the *Genotyping* section). From these recombinant F_3_ progenies, fixed recombinant lines were selected based on the marker genotypes. The DH population was developed using the maize pollination method as described by Thomas *et al.* (1997). For the validation of the newly developed MAS markers, a panel of 32 wheat lines was used which consisted of material ranging from F_1_ progeny to advanced generations (with *Lr32* plus additional Lr genes), *Lr32*-lacking susceptible check Neepawa, and an *Lr32* carrier (BW196R).

### Leaf rust testing

The leaf rust phenotyping of the parents, Thatcher and BW196R, DH population, F_2_ population, F_3_ progenies from the initial F_2_ population, and fixed recombinants derived from expanded F_2_ population was done with the urediospores of *Pt* race 12-3 MBDS at the seedling stage following the method described by McCallum and Seto-Goh (2003). Briefly, the plants were inoculated at the first leaf stage (∼7 day old) with urediospores of *Lr32* avirulent *Pt* race 12-3 MBDS suspended in light mineral oil (McCallum *et al.* 2021). After inoculation plants were kept in humidity-maintained chambers for 16 h at 18–20°C and subsequently transferred to the greenhouse which was kept at 20 ± 2°C. After 12–14 days plants were scored in the scale of 0–4 (0, 1, 2, 3, 4) where 0–2 were considered resistant and 3–4 considered susceptible, the symbols “+” and “−” were used to note pustule sizes that were larger or smaller than typically observed for a given infection type (McCallum *et al.* 2021).

### Genotyping and marker development

The DNA extraction of parents and mapping populations (DH, F_2_, and fixed recombinants) was done using a modified ammonium acetate method (Pallota *et al.* 2003). The DH population was genotyped using 90K iSelect SNP array (Wang *et al.* 2014) and 8 SSR markers, *barc128*, *barc135*, *barc376*, *cfd34*, *gwm2*, *gwm183*, *wmc43*, and *wmc539* reported in the Thomas *et al.* (2010). A linkage map was constructed using the software MapDisto 1.8.2 (Lorieux 2012) and genetic distances were calculated using the Kosambi mapping function (Kosambi 1943). After locating the *Lr32* region on the linkage map, the associated SNPs were converted into KASP markers (Tables 1 and 2). Total 17 KASP were developed from the 90K SNP array and genotyping was done according to the procedure described in Kassa *et al.* (2016). One SSR marker *Swh28* was developed from the *A. tauschii* pseudomolecules v4.0 gene-based region by using the software package Batchprimer3 (You *et al.* 2008) (Table 3). In addition, FASTA sequences of NBS-LRR gene regions from International Wheat Genome Sequencing Consortium (IWGSC) ReqSeq v1.0 were also used to develop the SSR markers, and out of which *Swh45* is polymorphic between parents (Table 3). The F_2_ population (*n* = 196) was genotyped with the 12 KASP developed in the DH population and 3 SSR markers *barc135*, *Swh28*, and *Swh45.* After that, codominant flanking DNA markers were selected and these markers were used for the screening of ∼2,000 F_2_ individuals to identify plants with a recombination event within the interval carrying *Lr32*. F_3_ progeny (minimum 16 progenies/family) from each recombinant F_2_ plant was tested with the flanking markers to select progeny fixed for recombination events and were genotyped with the remaining KASP markers.

**Table 1.** *Lr32* region-associated kompetitive allele-specific (KASP) PCR markers source, SNP name, primer sequence information.

Marker name	SNP name/gene ID	Primer name	Primer A1 (5ʹ–3ʹ)^a^	Inheritance
*Kwh142*	RFL_Contig2471_119	*kwh142-A1*	{Tail-1}GGGTCGAACACGTTGCTCCG	Codominant
		*kwh142-A2*	{Tail-2}GGGTCGAACACGTTGCTCCA	
		*kwh142-C*	CGAGCGCACAAGCCGAAGCAAT	
*Kwh147*	RAC875_c59977_246	*kwh147-A1*	{Tail-1}AAGCAACACAGATCTCCACCTCA	Codominant
		*kwh147-A2*	{Tail-2}AGCAACACAGATCTCCACCTCG	
		*kwh147-C*	GGTCGGTGTTGCCTCCTCGGT	
*Kwh148*	BobWhite_c16071_165	*kwh148-A1*	{Tail-1}AACGCCAAGATCGAAGCGTATTAC	Codominant
		*kwh148-A2*	{Tail-2}GAACGCCAAGATCGAAGCGTATTAT	
		*kwh148-C*	TCCAGTCCAGTACCGGTCTCGT	
*Kwh149*	D_contig34048_162	*kwh149-A1*	{Tail-1}ATTCCACATTGTGGCTTTCCAACCT	Codominant
		*kwh149-A2*	{Tail-2}CCACATTGTGGCTTTCCAACCC	
		*kwh149-C*	TCGAATACGATGACCCTGATGCCAT	
*Kwh152*	D_GDRF1KQ02F7Y6F_336	*kwh152-A1*	{Tail-1}CCTGTCGCACCGCAGCCAT	Codominant
		*kwh152-A2*	{Tail-2}CTGTCGCACCGCAGCCAC	
		*kwh152-C*	CCATTGTTTGATCTCTTTTTGCAGCGTT	
*Kwh340*	BS00093856_51	*kwh340-A1*	{Tail-1}GTTGAGTGGTATGCCGCCGC	Codominant
		*kwh340-A2*	{Tail-2}GGTTGAGTGGTATGCCGCCGT	
		*kwh340-C*	CATTTCTGTGGTGCGGCTGTGCAT	
*Kwh342*	D_contig09222_937	*kwh342-A1*	{Tail-1}CTTTCTGAATGTTACAAAGTATTTACATGTTA	Codominant
		*kwh342-A2*	{Tail-2}CTTTCTGAATGTTACAAAGTATTTACATGTTG	
		*kwh342-C*	GCTCAACCCTCAGCTGAAGCTGAA	
*Kwh343*	D_contig17344_388	*kwh343-A1*	{Tail-1}CAAACTGTTCGATGCTATGAATTTAGTG	Codominant
		*kwh343-A2*	{Tail-2}ATCAAACTGTTCGATGCTATGAATTTAGTA	
		*kwh343-C*	TGCAAAATTAACCCGCAAAACTCACGAT	
*Kwh345*	D_F5XZDLF01EIBB2_65	*kwh345-A1*	{Tail-1}GAGTTTAAAACAAGAACAGAGCAACAAA	Codominant
		*kwh345-A2*	{Tail-2}GAGTTTAAAACAAGAACAGAGCAACAAC	
		*kwh345-C*	GACAACATCACTCATGCAGTACTCCTT	
*Kwh346*	D_F5XZDLF02HWOJZ_227	*kwh346-A1*	{Tail-1}AACAGCAAGCTTTCTCTGGTCACAA	Codominant
		*kwh346-A2*	{Tail-2}CAGCAAGCTTTCTCTGGTCACAG	
		*kwh346-C*	GAAATATATGGATCAACTGCGAGCACTTT	
*Kwh349*	Excalibur_c11594_181	*kwh349-A1*	{Tail-1}GGCCCTGCCTATCCAACAAGAA	Codominant
		*kwh349-A2*	{Tail-2}GCCCTGCCTATCCAACAAGAG	
		*kwh349-C*	CCCACAGGAAAAAGTAAATCCAACGTATT	
*Kwh353*	Kukri_c28730_207	*kwh353-A1*	{Tail-1}TGCTCAACGGATGATCGCACAT	Codominant
		*kwh353-A2*	{Tail-2}GCTCAACGGATGATCGCACAC	
		*kwh353-C*	GCAGCTCTCTCTGTTGGATCYTCAA	
*Kwh355*	Kukri_c80364_374	*kwh355-A1*	{Tail-1}GTGTGTTGTCCAATTGCTGGTTGT	Codominant
		*kwh355-A2*	{Tail-2}GTGTTGTCCAATTGCTGGTTGC	
		*kwh355-C*	CTGTAGTGGTCTTCAAAGTCCTCCAA	
*Kwh357*	RAC875_c10628_1037	*kwh357-A1*	{Tail-1}GCCTGTTACTGCTGCCAAGACT	Codominant
		*kwh357-A2*	{Tail-2}CCTGTTACTGCTGCCAAGACG	
		*kwh357-C*	GCTCACCATTATCAGGTAGCCTTCAT	
*Kwh360*	RAC875_rep_c110663_1499	*kwh360-A1*	{Tail-1}ATCCCTTTACACCGTATGCTTTCG	Codominant
		*kwh360-A2*	{Tail-2}ATATCCCTTTACACCGTATGCTTTCA	
		*kwh360-C*	ATCCTCTCCGAAACATTTCTTCATTGCTT	
*Kwh362*	wsnp_Ex_c1602_3055066	*kwh362-A1*	{Tail-1}CAAACATGACTATGGCACGATAGAC	Codominant
		*kwh362-A2*	{Tail-2}CCAAACATGACTATGGCACGATAGAA	
		*kwh362-C*	GAACTGTGATATACTTTCCGTATCAGCAA	
*Kwh364*	BobWhite_c17617_133	*kwh364-A1*	{Tail-1}ACCAACGAGGAAACCATCGACG	Codominant
		*kwh364-A2*	{Tail-2}CACCAACGAGGAAACCATCGACA	
		*kwh364-C*	AGTTGGCCGCGCAGCCCGA	
*Kwh489*	BS00070468	*kwh489-A1*	{Tail-1}CCTTGTGGACAATTTCCTCTTTTGG	Dominant
		*kwh489-A2*	{Tail-2}CCCTTGTGGACAATTTCCTCTTTTGA	
		*kwh489-C*	CGTTGATTATTCAACAGCAGATGGGATTT	
*Kwh491*	BS00072718	*kwh491-A1*	{Tail-1}CCTTGTGGACAATTTCCTCTTTTGG	Dominant
		*kwh491-A2*	{Tail-2}CCCTTGTGGACAATTTCCTCTTTTGA	
		*kwh491-C*	CGTTGATTATTCAACAGCAGATGGGATTT	
*Kwh605*	BA00271713	*kwh605-A1*	{Tail-1}CACCAGAGTCATAATTTAAGTTGGAG	Codominant
		*kwh605-A2*	{Tail-2}CCACCAGAGTCATAATTTAAGTTGGAA	
		*kwh605-C*	CCTCAGAAAATGCACCTGGCAGTA	
*Kwh617*	BA00169625	*kwh617-A1*	{Tail-1}AGGCAAATGATACAGGTGCAGCT	Codominant
		*kwh617-A2*	{Tail-2}GGCAAATGATACAGGTGCAGCC	
		*kwh617-C*	CATTCCTCAGTTCTTGTTCTTGCAG	
*Kwh628*	BA00575742	*kwh628-A1*	{Tail-1}CGGGACGAGACTTGAGGAACC	Codominant
		*kwh628-A2*	{Tail-2}CGGGACGAGACTTGAGGAACT	
		*kwh628-C*	ACGTGGAGTATCTCCGCCGGA	
*Kwh645*	>AET3Gv20190400.18	*kwh645-A1*	{Tail-1}GCACAGAACAACTTGACTGGGC	Codominant
		*kwh645-A2*	{Tail-2}GGCACAGAACAACTTGACTGGGT	
		*kwh645-C*	CACCGACAGCAATGTCAAATTTTGTAGAA	
*Kwh646*	>AET3Gv20190400.18	*kwh646-A1*	{Tail-1}CAGGGTCACTCATGCTACTAGTG	Codominant
		*kwh646-A2*	{Tail-2}AACAGGGTCACTCATGCTACTAGTT	
		*kwh646-C*	CTTTGAGCTTCCTGTGGAGTAACCAA	
*Kwh662*	>AET3Gv20200600.2	*kwh662-A1*	{Tail-1}GCGTGCGAGATCGGCGTC	Codominant
		*kwh662-A2*	{Tail-2}GCGTGCGAGATCGGCGTG	
		*kwh662-C*	GCACGATGTTGCGGTGGCGCA	
*Kwh721*	BA00024318	*kwh721-A1*	{Tail-1}CGTCTGTAGAATACAAGGTTCTC	Dominant
		*kwh721-A2*	{Tail-2}CTCGTCTGTAGAATACAAGGTTCTG	
		*kwh721-C*	CCTCCACATGTGTGTTACAAAATTACAAAT	
*Kwh722*	BA00186143	*kwh722-A1*	{Tail-1}ACCATGGATCCTACCAAAGAGGA	Codominant
		*kwh722-A2*	{Tail-2}CCATGGATCCTACCAAAGAGGG	
		*kwh722-C*	GCGGCTATTGTTCGACAACTGCTAA	
*Kwh727*	BA00078867	*kwh727-A1*	{Tail-1}CTTCCCCTTCCTTTCTGTTT	Dominant
		*kwh727-A2*	{Tail-2}GCTCTTCCCCTTCCTTTCTGTTC	
		*kwh727-C*	CAGAGGTGTGTATGTGGTGATGGAT	

a Tail-1 (FAM tail-GAAGGTGACCAAGTTCATGCT), Tail-2 (VIC tail-GAAGGTCGGAGTCAACGGATT)

**Table 2.** Sequence information for the SSR markers developed from the Chinese spring RefSeq v2.1 and *A. tauschii* pseudomolecules v5.0.

Marker name	Forward	Reverse
*Swh28*	AGTCGTCCTGGCTTACGTGT	CGAAACGCACCTTGCTTTAT
*Swh45*	AGCGGCTGTAGCTTTTGTTC	TGCATGAATGTTTGGTCCAG

**Table 3.** Coordinates of Kompetitive allele-specific (KASP) PCR and the SSR markers source sequences on the Chinese spring RefSeq v2.1 and *A. tauschii* pseudomolecules v5.0.

Marker name	SNP name/gene ID	Source	IWGSC RefSeq v2.1	*Aegilops tauschii* pseudomolecules v5.0
*Kwh142*	RFL_Contig2471_119	90K Wheat SNP^a^	17416838–17416738	17822798–17822698
*Kwh489*	BS00070468	CerealsDB-KASP^b^	18794498–18794398	19599880–19599780
*Kwh491*	BS00072718	CerealsDB-KASP	18794498–18794398	19599880–19599780
*Kwh721*	BA00024318	Axiom 35K and 820K*c*	19907918–19907818	20595051–20594951
*Kwh722*	BA00186143	Axiom 35K and 820K	19960762–19960862	20647473–20647573
				20640902–20641002
				20656768–20656868
*Kwh727*	BA00078867	Axiom 35K and 820K	21829292–21829390	23302561–23302659
*Kwh340*	BS00093856_51	90K Wheat SNP	22049657–22049557	23435820–23435720
*Kwh149*	D_contig34048_162	90K Wheat SNP	24039549–24039298	25780968–25781219
				26231058–26230808
*Swh28*		*Aegilops tauschii* pseudomolecules v5.0	—	27076467–27076484
		gene region		
*Swh45*		IWGSC RefSeq_NB-LRR region	26290206–26294113	—
*Kwh147*	RAC875_c59977_246	90K Wheat SNP	28724554–28724454	30772352–30772252
				30783260–30783160
*Kwh346*	D_F5XZDLF02HWOJZ_227	90K Wheat SNP	31777459–31777708	34568768–34569017
*Kwh360*	RAC875_rep_c110663_1499	90K Wheat SNP	32277430–32277331	—
*Kwh364*	BobWhite_c17617_133	90K Wheat SNP	33167119–33167035	36372653–36372569
*Kwh357*	RAC875_c10628_1037	90K Wheat SNP	36693579–36693479	38119477–38119377
*Kwh148*	BobWhite_c16071_165	90K Wheat SNP	36693749–36693849	38119647–38119747
*Kwh342*	D_contig09222_937	90K Wheat SNP	37167341–37167092	38613914–38613665
*Kwh349*	Excalibur_c11594_181	90K Wheat SNP	37846513–37846613	39264726–39264823
*Kwh345*	D_F5XZDLF01EIBB2_65	90K Wheat SNP	39445515–39445330	40921768–40921582
*Kwh353*	Kukri_c28730_207	90K Wheat SNP	43918204–43918108	45744256–45744160
*Kwh605*	BA00271713	Axiom 35K and 820K	44114802–44114732	46068489–46068419
*Kwh645*	>AET3Gv20190400.18	*Aegilops tauschii* pseudomolecules v4.0^d^	45121378–45121531	47128503–47128656
*Kwh646*	>AET3Gv20190400.18	*Aegilops tauschii* pseudomolecules v4.0	45121532–45121779	47128657–47128904
*Kwh617*	BA00169625	Axiom 35K and 820K	45122722–45122652	47129847–47129777
*Kwh362*	wsnp_Ex_c1602_3055066	90K Wheat SNP	—	480327588–480327388
*Kwh628*	BA00575742	Axiom 35K and 820K	46020254–46020324	49080031–49080101
*Kwh662*	>AET3Gv20200600.2	*Aegilops tauschii* pseudomolecules v4.0	46675509–46675694	49760125–49760310
*Kwh152*	D_GDRF1KQ02F7Y6F_336	90K Wheat SNP	47438552–47438794	50412774–50413021
*Kwh343*	D_contig17344_388	90K Wheat SNP	47742563–47742314	50841817–50841568
*Kwh355*	Kukri_c80364_374	90K Wheat SNP	47743405–47743326	50842656–50842580

a Wang *et al.* (2014).

b Wilkinson *et al.* (2012).

c Allen *et al.* (2017).

d Luo *et al.* (2017).

e No hit.

To increase the resolution of the *Lr32* spanning genetic region, an additional 11 KASP markers were developed or selected from the CerealsDB database, 35K and 820K Axiom R SNPs, and *A. tauschii* (accession AL8/78) pseudomolecules v4.0 (Tables 1 and 2). First, the flanking KASP markers source sequences were aligned against the IWGSC ReqSeq v1.0 via using the basic local alignment search tool (BLAST) (Altschul *et al.* 1997) and the *Jbrowse* portal was used to explore the region. Within that region, 2 SNPs (BS00070468 and BS00072718) were selected and associated KASP markers *Kwh489* and *Kwh491* (Tables 1 and 2) were used for genotyping the fixed recombinant lines (CerealsDB database; Wilkinson *et al.* 2012). Within that region, additional 35K and 820K Axiom R SNPs were also selected to develop KASP markers (Allen *et al.* 2017). Total 6 KASP markers, *Kwh605*, *Kwh617*, *Kwh628*, *Kwh721*. *Kwh722*, and *Kwh727*, were developed from the Axiom SNPs and used to genotype the fixed recombinants (Tables 1 and 2). Moreover, the flanking KASP markers source sequences were also used to select the *A. tauschii* (accession AL8/78) pseudomolecules v4.0 (Luo *et al.* 2017) gene regions. Out of which, LRR motif-containing gene regions were selected and BLASTed against the IWGSC RefSeq v1.0 assembly. After aligning the FASTA sequence from both assemblies, SNPs were identified and 3 KASP markers, *Kwh645*, *Kwh646*, and *Kwh662*, were manually developed by using the Primer3 software package (Ye *et al.* 2012). The source sequences of KASP and SSR markers were used to determine the physical coordinates on the *A. tauschii* pseudomolecules v4.0 and IWGSC ReqSeq v2.1. The mode of inheritance (dominant/codominant) of the markers developed in the current study was determined on the F_2_ population. To validate these markers a genetic analysis was conducted with F_1_ and prebreeding germplasm carrying *Lr32* (Supplementary Table 1). The Aet_v4.0 and IWGSC ReqSeq v2.1 reference genomes data were used to extract the disease resistance candidate genes between flanking markers *Kwh142* and *Kwh355*.

## Results

**Fig. 1.**
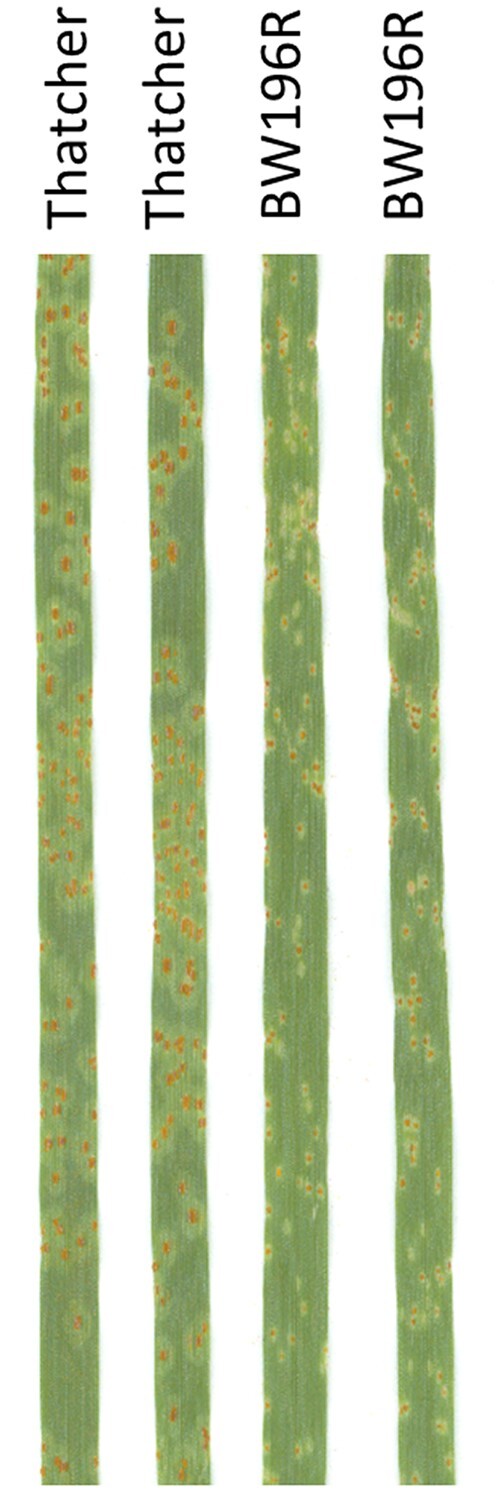
Seedling stage leaf rust infection type (IT) on the *Lr32* DH mapping populations parents Thatcher (IT = 3) and BW196R (IT =; 1−) 14th day postinoculation of the *Pt* race MBDS.

**Fig. 2.**
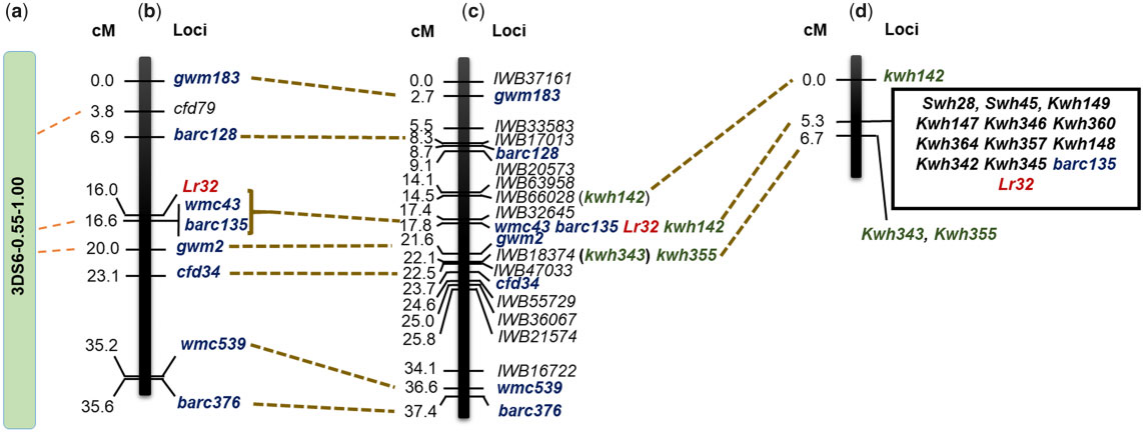
a) The chromosome 3DS deletion Bin6. b) The SSR markers based chromosome arm 3DS linkage map spanning *Lr32* developed in the DH population (Thomas *et al.* 2010). c) The *Lr32* associated chromosome arm 3DS 90K SNP’s linkage map developed in the Thatcher × BW196R DH population. d) The 3DS linkage map developed in the Thatcher × BW196R F_2_ population (*n* = 196) by using 12 KASP and 3 SSR markers. Common SSR markers between 3DS map developed by Thomas *et al.* (2010) and current study were shown in *Blue fonts* and KASP markers developed from the 90K SNPs were represented with the *green fonts*.

The leaf rust screening showed that the susceptible parent Thatcher had IT 3 and the resistant parental line BW196R had IT 1− (Fig. 1). The DH population phenotypic ratio fitted that expected for a single gene (109R:134S; *χ*^2^ = 2.25, *P* = 0.11). The initial F_2:3_ population had 51 resistant (HR), 96 segregating (hetero), and 49 susceptible (HS) families which also fitted a single gene ratio (χ^2^ = 0.12, *P* = 0.94). The DH and F_2:3_ population IT scores ranged between 1− and 3 (HR = 1−/12/12, hetero = 2− to 3−, HS = 3/3+) (Supplementary Files 1 and 2). Linkage mapping in the DH population using 90K SNP markers and SSR markers developed for the *Lr32* region resulted in a genetic map of chromosome arm 3DS that spanned 37.4 cM and consisted of 39 SNP and 8 SSR markers (Fig. 2 and Supplementary Files 1 and 2). *Lr32* was mapped at position 17.8 cM and cosegregated with SSRs *wmc43* and *barc135*. In addition, a total of 24 SNP markers cosegregated with *Lr32* (Supplementary Files 1 and 2). This region was flanked by SNPs *IWB32645* and *IWB18374* (Fig. 2). Genetic analysis conducted on the F_2_ population with 12 KASP and 3 SSR markers developed a chromosome arm 3DS linkage map of 6.73 cM (Fig. 2).

**Fig. 3.**
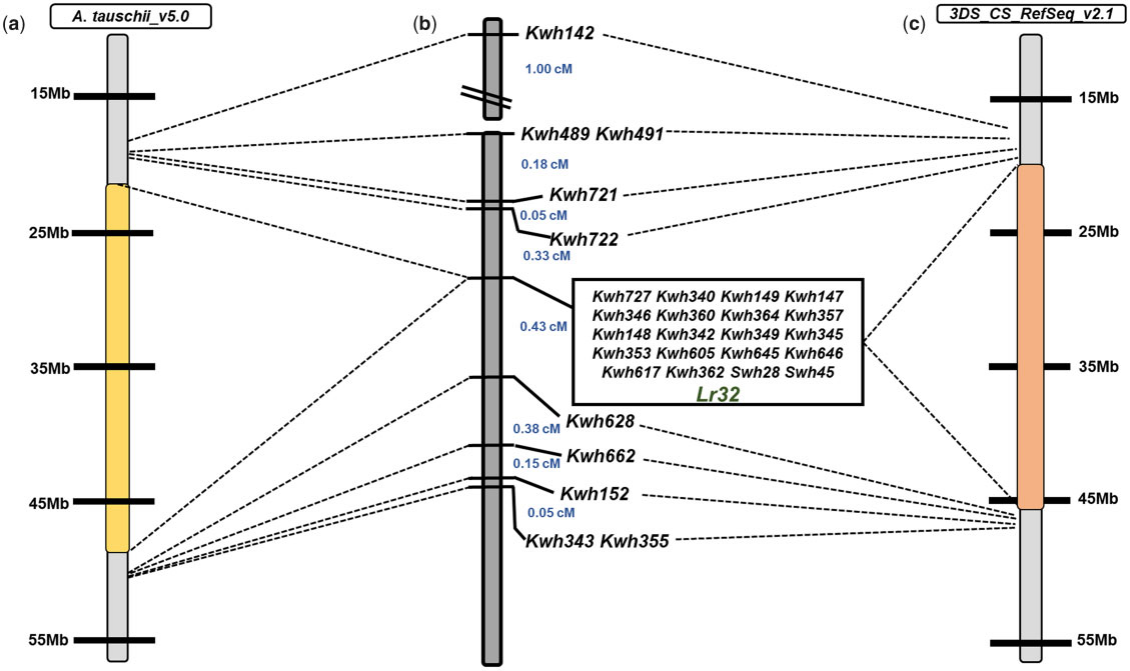
Collinearity of *Lr32* high resolution genetic region-associated KASP markers (b) on the *Aegilops tauschii* (Aet v 5.0) (a) and 3DS Chinese spring reference (Refseq v2.1) genomes. The high-resolution mapping was done by using 106 fixed recombinants.

The KASP markers *Kwh142* and *Kwh355* positioned at 14.5 and 22.1 cM on the chromosome 3DS DH linkage map were selected as the flanking markers and used for screening the ∼2,000 F_2_ progenies (Fig. 2). Selected F_2_ progeny were used to generate F_3_ progeny. Based on genotypic and phenotypic screening of these F_3_ progenies, 106 fixed recombinants (i.e. both homologs carried the same recombination event between the flanking markers with no heterozygous alleles for the flanking markers) were identified (Fig. 3). Further genetic analysis was done on these 106 fixed recombinants with the remaining 26 KASP and 2 SSR markers were developed from 90K SNP chip, Axiom SNPs, *A. tauschii* gene-based regions and CerealsDB database (Fig. 3 and Tables 1 and 2). The overall genomic analysis conducted on the fixed recombinants fine-mapped *Lr32* to a region spanning 2.57 cM between the flanking markers. In the fine mapping population, a total of 18 KASP and 2 SSR markers cosegregated with *Lr32* and were flanked by KASP markers *Kwh 722* and *Kwh 638* (Supplementary File 3). Mapping of KASP markers source sequences on reference genomes showed that the *Lr32* region is collinear in both diploid and hexaploid genome assemblies. Total 11 and 10 candidate genes belonging to the gene’s classes typical of disease resistance genes were identified between the flanking markers *Kwh142* and *Kwh355* in the IWGSC RefSeq v2.1 and *A. tauschii* pseudomolecules v4.0. reference genomes, respectively (Table 4).

**Table 4.** Candidate genes between flanking markers *Kwh142* and *Kwh355* identified in reference genome assemblies of *A. tauschii* pseudomolecules v4.0 and International Wheat Genome Sequencing Consortium (IWGSC) RefSeq v2.1.

Gene list	*Aegilops tauschii* pseudomolecules v4.0 coordinates	IWGSC RefSeq v2.1 coordinates	Gene function
LOC109770626	371107079–371130152	—	Leaf rust 10 disease resistance locus receptor-like PROTEIN KINASE-like 2.8% 2C transcript variant ×1
LOC109770629	371186179–371191403	—	Leaf rust 10 disease resistance locus receptor-like PROTEIN KINASE-like 2.7
LOC109770630	371356207–371376463	—	Leaf rust 10 disease resistance locus receptor-like PROTEIN KINASE-like 1.2% 2C transcript variant ×2
LOC109786043	403287941–403288597	—	Putative disease resistance protein RGA3
LOC109786041	403289550–403292234	—	Disease resistance protein RGA2
LOC109786040	403294816–403296642	—	Disease resistance protein RGA2
LOC109757481	431756637–431759708	—	Disease resistance protein RPM1-like
LOC109749583	439761646–439765270	—	Disease resistance protein RPS2
LOC109779823	442696038–442705114	—	Disease resistance RPP13-like protein 4%2C transcript variant ×4
LOC109779825	442700026–442701359	—	Probable disease resistance protein At5g45440
TraesCS3D03G0659400.1	—	396867186–396868224	NBS–LRR-like resistance protein
TraesCS3D03G0659900.1	—	396875344–396875847	Disease resistance protein RGA2
TraesCS3D03G0683400.1	—	413254998–413257949	Disease resistance protein (TIR–NBS class)
TraesCS3D03G0697900.1	—	421489316–421489550	Disease resistance protein (TIR–NBS–LRR class) family
TraesCS3D03G0705600.1	—	425470383–425473025	NBS–LRR disease resistance protein
TraesCS3D03G0721700.1	—	433489667–433492396	CC–NBS–LRR family disease resistance protein
TraesCS3D03G0728800.1	—	436220239–436221414	NBS–LRR disease resistance protein
TraesCS3D03G0728900.1	—	436222190–436223924	Disease resistance protein
TraesCS3D03G0737800.1	—	440586174–440589527	NBS–LRR resistance protein, putative
TraesCS3D03G0790700.1	—	469421914–469422111	Disease resistance protein (TIR–NBS–LRR class) family
TraesCS3D03G0801200.1	—	476629268–476630671	Nematode resistance protein-like HSPRO2

## Discussion

The phenotyping results showed that the DH and initial F_2_ populations segregated for 1 gene. The collinearity of SSR and KASP markers in the DH and F_2_ mapping populations and their corresponding physical order on reference genomes demonstrate the marker order was robust (Figs. 1 and 3). In addition, large numbers of 90K SNP/KASP markers cosegregating with the *Lr32* resistance phenotype in the 3 populations (DH, F_2_, and fixed recombinants) analyzed in the current study indicates that *Lr32* region is associated with a linkage block with recombination restriction. Physical mapping of the SNP source sequences on the *A. tauschii* pseudomolecules v4.0 and IWGSC ReqSeq v2.1 reference genomes showed that the *Lr32* region spanned about 34–36 Mb between the flanking markers *Kwh142* and *Kwh355*, which clearly showed that the physical size of these segments is large (11–13 Mb/cM) (Fig. 3). The cosegregating markers with *Lr32* phenotype represent 26–28 Mb on both reference genomes (Fig. 3). These results explained the limitation to further increase the resolution of the *Lr32* region. In addition, it also indicates a lower recombination frequency associated with alien R genes. Among the candidate genes identified from the reference genomes, NLR is the most abundant one. Besides that Protein Kinase and RGA types genes were also identified. Both classes of NLR genes, Toll-interleukin receptor type (TIR) and N-terminal coiled-coil (CC) were identified. Whereas, to date only CC–NBS–LRR has been identified in wheat for disease resistance (reviewed in Md. Hatta *et al.* 2019).

**Fig. 4.**
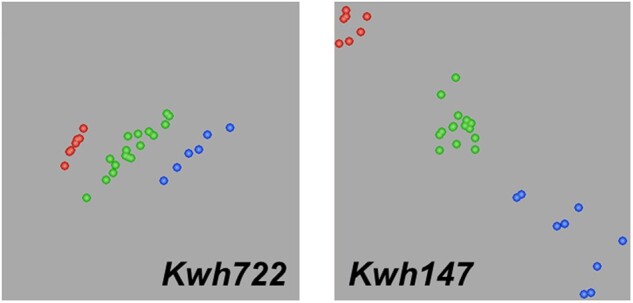
The validation analysis done on the F_1_ and advanced multigene lines with *Lr32*-region functional molecular markers *Kwh 147* and *Kwh 722*.

The validation analysis done with the F_1_ and advanced prebreeding lines showed that the 2 KASP markers *Kwh147* and *Kwh722* are codominant and able to differentiate between plants carrying *Lr32* in the heterozygous/homozygous states (Fig. 4). Marker *Kwh340* also detects the presence of the *Lr32*-resistant allele; however, clusters are not well differentiated. These markers should be useful for the selection of *Lr32* in wheat breeding programs. Pyramiding *Lr32* with other effective genes such as adult–plant resistance genes *Lr34*, *Lr46* or *Lr67* should help to delay the evolution of *Lr32* virulent *P. triticina* isolates. To date virulence to *Lr32* has not been detected in Canada (McCallum *et al.* 2021), although there is a report for virulence in South Africa (Pretorius and Bender 2010). The absence of virulence to *Lr32* in Canada may be due to the fact that this gene has not been deployed in a commercial wheat cultivar to drive evolution of virulence in the pathogen population. The resistance gene *Lr67* was recently shown to have a significant interaction with *Lr32* when the 2 genes were in combination in a population that segregated for these 2 genes in the Thatcher background (McCallum and Hiebert 2022). The interaction between *Lr34* and *Lr32* was not significant in a population that segregated for both these genes; however, lines with both genes were more resistant than lines with either-gene alone. Using the molecular markers developed in this study *Lr32* can be successfully incorporated into wheat lines, such as Carberry with *Lr34*, *Lr46*, and other resistance genes, that already have a good base of genetic resistance (Bokore *et al.* 2022). Deploying *Lr32* in isolation would likely lead to the evolution of virulence on *Lr32* as was seen for *Lr21*, a similar resistance gene also derived from *A*. *tauschii*, both in the USA (Kolmer and Anderson 2011) and Canada (McCallum *et al.* 2017). In conclusion, the development of these new functional markers will accelerate integration of *Lr32* into the breeding lines and will be helpful in MAS to develop the future wheat cultivars with durable resistance.

## Supplementary Material

jkac274_Supplemental_File_1

jkac274_Supplemental_File_2

jkac274_Supplemental_File_3

jkac274_Supplemental_Table_1

jkac274_Supplemental_Material_Legend

## Data Availability

Seed is available upon request. The authors affirm that all data necessary for confirming the conclusions of the article are present within the article, figures, and tables. Genotypic and phenotypic data available in supplementary files. Supplemental material is available at G3 online.
