# Supplementary material for: Fine mapping and marker development for the wheat leaf rust resistance gene Lr32
Source: G3 (Bethesda). 2022 Oct 18;13(2):jkac274. doi: 10.1093/g3journal/jkac274 (PMC9911047; doi:10.1093/g3journal/jkac274)
Supplement: jkac274_Supplemental_Table_1 [file jkac274_supplemental_table_1.docx]

| **Supplementary Table 1**. Validation of the kompetitive allele specific PCR (KASP) markers on the pre-breeding germplasm carrying *Lr32* | | | | |  |
| --- | --- | --- | --- | --- | --- |
| Plant # | Description | Traits | *Kwh147^1^* | *Kwh722^1^* |  |
| CX880 | F_1_ | Lr22a, Lr32, Lr52 & Lr70 | H | H |  |
| CX881 | F_1_ | Lr22a, Lr32, Lr52 & Lr70 | H | H |  |
| CX882 | F_1_ | Lr22a, Lr32, Lr52 & Lr70 | H | H |  |
| CX883 | F_1_ | Lr22a, Lr32, Lr52 & Lr70 | H | H |  |
| CX884 | F_1_ | Lr22a, Lr32, Lr52 & Lr70 | H | H |  |
| CX885 | F_1_ | Lr22a, Lr32, Lr52 & Lr70 | H | H |  |
| CX886 | F_1_ | Lr22a, Lr32, Lr52 & Lr70 | H | H |  |
| CX887 | F_1_ | Lr22a, Lr32, Lr52 & Lr70 | H | H |  |
| CX888 | F_1_ | Lr22a, Lr32, Lr52 & Lr70 | H | H |  |
| CX889 | F_1_ | Lr22a, Lr32, Lr52 & Lr70 | H | H |  |
| CX890 | F_1_ | Lr22a, Lr32, Lr52 & Lr70 | H | H |  |
| CX891 | F_1_ | Lr22a, Lr32, Lr52 & Lr70 | H | H |  |
| CX892 | F_1_ | Lr22a, Lr32, Lr52 & Lr70 | H | H |  |
| CX893 | F_1_ | Lr22a, Lr32, Lr52 & Lr70 | H | H |  |
| CX894 | F_1_ | Lr22a, Lr32, Lr52 & Lr70 | H | H |  |
| CX895 | F_1_ | Lr22a, Lr32, Lr52 & Lr70 | H | H |  |
| CX896 | CN1669/Lr70/BW1050*2 | Lr22, Lr32 & Lr52 | A | A |  |
| CX897 | CN1669/Lr70/BW1050*2 | Lr22, Lr32 & Lr52 | A | A |  |
| CX898 | CN1669/Lr70/BW1050*2 | Lr22, Lr32 & Lr52 | A | A |  |
| CX899 | CN1669/Lr70/BW1050*2 | Lr22, Lr32 & Lr52 | A | A |  |
| CX900 | CN1669/Lr70/BW1050*2 | Lr22, Lr32 & Lr52 | A | A |  |
| CX901 | CN1669/Lr70/BW1050*2 | Lr22, Lr32 & Lr52 | A | A |  |
| CX902 | Neepawa^2^ | Lacks Lr22a and Lr32 | B | B |  |
| CX903 | RL6044 | RL6044-Lr22a | B | B |  |
| CX904 | BW196R-2 | Lr32 | A | A |  |
| CX905 | RL6058 | Thatcher-Lr34 | B | B |  |
| CX906 | Ku 3198 | Lr70, Lr52? | B | B |  |
| CX907 | Hy 644 | Lacks Lr70 | B | B |  |
| CX908 | Tc-Lr52 | Lr52 | B | B |  |
| CX909 | BW410 | Lr22a | B | B |  |
| CX910 | BW1068 | Lr21, Lr23, Lr34, Sm1 & FHB1 | B | B |  |
| CX911 | BW1068 | Lr21, Lr23, Lr34, Sm1 & FHB1 | B | B |  |
| ^1^Scoring: A = Lr32 allele, B = Lr32 absent, H = Hetero for Lr32 loci  ^2^Neepawa: Campbell AB. 1970. Neepawa hard red spring wheat. Canadian Journal of Plant Science. 50:752-753.  https://doi.org/10.4141/cjps70-143 | | | | | |
