# Supplementary material for: Fine mapping and marker development for the wheat leaf rust resistance gene Lr32
Source: G3 (Bethesda). 2022 Oct 18;13(2):jkac274. doi: 10.1093/g3journal/jkac274 (PMC9911047; doi:10.1093/g3journal/jkac274)
Supplement: jkac274_Supplemental_Material_Legend [file jkac274_supplemental_material_legend.docx]

**SUPPLEMENTARY FILES**

**Supplementary file 1:** The chromosome 3DS linkage map developed in the Thatcher × BW196R DH population.

**Supplementary file 2:** Genotypic and phenotypic data of the Thatcher × BW196R F_2_ population.

**Supplementary file 3**: The 106 fixed recombinants genotypic and phenotypic score for high-resolution mapping

**Supplementary Table 1**. Validation of the kompetitive allele specific PCR (KASP) markers on the pre-breeding germplasm carrying *Lr32*
